# Independent Losses of Visual Perception Genes *Gja10* and *Rbp3* in Echolocating Bats (Order: Chiroptera)

**DOI:** 10.1371/journal.pone.0068867

**Published:** 2013-07-18

**Authors:** Bin Shen, Tao Fang, Mengyao Dai, Gareth Jones, Shuyi Zhang

**Affiliations:** 1 Institute of Molecular Ecology and Evolution, Institutes for Advanced Interdisciplinary Research, East China Normal University, Shanghai, China; 2 School of Biological Sciences, University of Bristol, Bristol, United Kingdom; Texas A&M University, United States of America

## Abstract

A trade-off between the sensory modalities of vision and hearing is likely to have occurred in echolocating bats as the sophisticated mechanism of laryngeal echolocation requires considerable neural processing and has reduced the reliance of echolocating bats on vision for perceiving the environment. If such a trade-off exists, it is reasonable to hypothesize that some genes involved in visual function may have undergone relaxed selection or even functional loss in echolocating bats. The Gap junction protein, alpha 10 (Gja10, encoded by *Gja10* gene) is expressed abundantly in mammal retinal horizontal cells and plays an important role in horizontal cell coupling. The interphotoreceptor retinoid-binding protein (Irbp, encoded by the *Rbp3* gene) is mainly expressed in interphotoreceptor matrix and is known to be critical for normal functioning of the visual cycle. We sequenced *Gja10* and *Rbp3* genes in a taxonomically wide range of bats with divergent auditory characteristics (35 and 18 species for *Gja10* and *Rbp3*, respectively). Both genes have became pseudogenes in species from the families Hipposideridae and Rhinolophidae that emit constant frequency echolocation calls with Doppler shift compensation at high-duty-cycles (the most sophisticated form of biosonar known), and in some bat species that emit echolocation calls at low-duty-cycles. Our study thus provides further evidence for the hypothesis that a trade-off occurs at the genetic level between vision and echolocation in bats.

## Introduction

The brain can consume up to 20% of circulating oxygen and glucose and overall brain size is constrained [1], [2]. Hence Harvey and Krebs (1990) [3] suggested that specialized enlargement of one area of the brain may be associated with reduction in size of another. Such trade-offs may be especially prominent in animals with specialized sensory modalities such as echolocation. In bats that use laryngeal echolocation brain areas such as the auditory cortex and the inferior colliculus are disproportionately large in volume, while in non-echolocating Old World fruit bats visual and olfactory brain areas are relatively enlarged [4].

Trade-offs in the relative sizes of brain regions will ultimately be determined by genetic mechanisms. Indeed, evidence for trade-offs in relative investment in different sensory modalities is emerging. A molecular evolutionary study of the short-wavelength opsin gene (*Sws1*) in bats [5] showed how insertions/deletions (indels) and stop codon mutations rendered the gene non-functional in bats using high-duty-cycle echolocation (i.e., species that spend >30% of time calling) [6]. Bats using high-duty-cycle echolocation emit long constant frequency (CF) calls and compensate for Doppler shifts induced by their own movement in flight, can thereby separate calls and echoes in the frequency domain [7] and have the most sophisticated echolocation known [8]. Thus, it is reasonable to hypothesize that more genes involved in visual function may have undergone relaxed selection or even functional loss in echolocating bats, especially in bats that use high-duty-cycle echolocation.

In this study, we focus on two visual perception genes, the *Gja10* gene encoding Gap junction protein, alpha 10 (Gja10) and the *Rbp3* gene encoding interphotoreceptor retinoid-binding protein (Irbp).

The retinal horizontal cells exhibit a significantly larger receptive field than predicted from individual dendritic fields by electrical coupling of cells to each other via gap junctions [9]. The *Gja10* gene is expressed abundantly in mammalian retinal horizontal cells [10], [11], [12]. The largest part of the Gja10 protein is encoded by exon 2 of *Gja10* gene (480 aa, ∼97.6%), and its remaining 12 amino acids (∼2.4%) are encoded by exon 3 via functional splicing during transcription [11], [12]. The deletion of *Gja10* in mice caused significant reduction in the size of the horizontal cell receptive field, indicating a pivotal role of Gja10 in horizontal cell coupling [13]. The interphotoreceptor retinoid-binding protein (Irbp, encoded by the *Rbp3* gene) is mainly expressed in the interphotoreceptor matrix (IPM) [14], where it is widely known to facilitate both the removal of all-*trans*-retinal from, and the delivery of 11-*cis*-retinal to, photoreceptors [15]. *Rbp3* knockout (*Rbp3* ^−/−^) mice display impaired transportation of 11-*cis*-retinal from retinal pigment epithelium to photoreceptors, degenerations of both cones and rods and exaggerated eye growth [16], [17], [18], [19], indicating an important role for *Rbp3* in normal retinal function. The *Rbp3* gene comprises four exons, among which the first exon (3054 bp, 1018 aa) encodes almost 81.6% of the Irbp protein [20]. Moreover the nucleotide sequence of the first exon is widely used as a genetic maker in phylogenetic studies [21], [22], [23], [24], [25].

Studies have reported that these two genes have became pseudogenes in the naked mole rat *Heterocephalus glaber* (both *Gja10* and *Rbp3*) [26] and the southern marsupial mole *Notoryctes typhlops* (*Rbp3*) [25] which are presumed to be parallel genetic changes corresponding to phenotypic degeneration of their vision. We therefore hypothesized that these two visual perception genes may also be targets for pseudogenization in echolocating bats in relation to the trade-off between vision and echolocation.

To test our hypothesis, we sequenced the partial coding sequences of *Gja10* and *Rbp3* from a taxonomically wide range of bats with and without laryngeal echolocation, and studied molecular evolutionary patterns of these two genes in bats.

## Materials and Methods

### Ethics Statement

We neither sampled nor killed any bats for this work. The wing membrane biopsies of bat species used in this study were taken from archive material collected and stored in 100% ethanol over the last decade in our lab [5], [27].

### Taxonomic Coverage

We sequenced the largest part of exon 2 sequences of *Gja10* (∼1200 bp) from 35 bat species covering 11 of the 17 extant chiropteran families, including six species from the family Pteropodidae (*Cynopterus sphinx*, *Rousettus leschenaultii*, *R. aegyptiacus, Eonycteris spelaea*, *Pteropus giganteus* and *Dobsonia viridis*), five from the family Rhinolophidae (*Rhinolophus ferrumequinum*, *R. pusillus*, *R. sinicus*, *R. affinis* and *R. pearsonii*), five from the family Hipposideridae (*Hipposideros cineraceus*, *H. armiger*, *H. pratti*, *H. pomona* and *Aselliscus stoliczkanus*), two from the family Megadermatidae (*Megaderma lyra* and *M. spasma*), one from the family Rhinopomatidae (*Rhinopoma hardwickii*), two from the family Mormoopidae (*Mormoops megalophylla* and *Pteronotus davyi*), six from the family Phyllostomidae (*Desmodus rotundus*, *Artibeus jamaicensis*, *A. lituratus*, *Leptonycteris yerbabuena*, *Anoura geoffroyi* and *Carollia perspicillata*), three from the family Vespertilionidae (*Scotophilus kuhlii*, *Myotis ricketti* and *Murina leucogaster*), one from the family Miniopteridae (*Miniopterus fuliginosus*), two from the family Molossidae (*Tadarida brasiliensis* and *T. plicata*) and two from the family Emballonuridae (*Taphozous melanopogon* and *Emballonura raffrayana*).

We also sequenced part of exon 1 in *Rbp3* (∼2000 to 2500 bp) from 18 bat species covering 9 chiropteran families, including three species from the family Pteropodidae (*C. sphinx*, *R. leschenaultii* and *E. spelaea*), four from the family Rhinolophidae (*R. luctus*, *R. pearsonii*, *R. pusillus* and *R. sinicus*), two from the family Hipposideridae (*H. armiger* and *H. pratti*), two from the family Megadermatidae (*M. lyra* and *M. spasma*), three from the family Mormoopidae (*M. megalophylla*, *P. davyi* and *P. parnellii*), one from the family Phyllostomidae (*A. jamaicensis*), one from the family Vespertilionidae (*Pipistrellus abramus*), one from the family Molossidae (*T. brasiliensis*) and one from the family Emballonuridae (*E. raffrayana*). All new sequences were deposited in GenBank and accession numbers are KC211187-KC211221 for *Gja10* and KC211222-KC211239 for *Rbp3*.

For phylogenetic reconstruction analyses, we also obtained available published *Gja10* and *Rbp3* sequences of five other mammal species from GenBank as outgroups. For the *Gja10* gene: *Homo sapiens* (NM_032602), *Mus musculus* (NM_010289), *Rattus norvegicus* (NM_001173508), *Bos taurus* (XM_001787431) and *Canis familiaris* (XM_003639398). For the *Rbp3* gene: *Homo sapiens* (NM_002900), *Mus musculus* (NM_015745), *Rattus norvegicus* (NM_001191832), *Bos taurus* (NM_174164) and *Canis familiaris* (XM_546201). The detailed information for all species, accession numbers and sequence lengths are listed in Table S1 for *Gja10* and *Rbp3*.

### Isolation, Amplification and Sequenceing

We isolated genomic DNA using DNeasy Blood & Tissue Kit (Qiagen) from wing membrane biopsies of the studied bat species that were collected and stored in 100% ethanol. For the *Gja10* gene, a pair of primers F (5′-CAG CCA GGT TGC AAC AAT ATC TG-3′) and R (5′- CT TAC CAT TGA TGT TCT GTG CCC A-3′) were designed based on the incomplete *Gja10* coding sequence of the bat species *Pteropus vampyrus* obtained from the Ensembl database (http://www. ensembl.org/) to amplify an extensive portion of exon 2 sequences of the gene in 35 bat species (Figure S1A). For the *Rbp3* gene, several pairs of primers were designed to amplify part of exon 1 sequences in 18 bat species. For species from the family Pteropodidae, Rhinolophidae and Hipposideridae, two pairs of primers F1 (5′-ATG ACA AGA GAA TGG GCC CTG CTC-3′) and R1 (5′-TG GAA AAC GGA GTC CAC TAG GGC-3′) and F2 (5′-AC GAT CTG GTC ACT AAG CTC AAC G-3′) and R2 (5′-AT CAG GAT GTA GAG GTC CTT GTG G-3′) were designed based on the incomplete *Rbp3* coding sequence of *P. vampyrus* obtained from the Ensembl database to amplify two overlapping fragments of partial *Rbp3* exon 1 sequences (∼1300 and 1400 bp, respectively) (Figure S1B). Then these two overlapping fragments were assembled together to obtain part of the *Rbp3* exon 1 sequences (∼2500 bp). For species from the family Megadermatidae and Vespertilionidae, a pair of primers F3 (5′-AGC CAG GAG GTG GTG AGC AAG TT-3′) and R3 (5′-GG AAT CTG GGC TGT CTT CAG GTG T-3′) were designed based on the incomplete *Rbp3* coding sequence of *Myotis lucifugus* obtained from Ensembl database to amplify part of *Rbp3* exon 1 sequences (∼2500 bp) (Figure S1B). Finally, for the remaining bat species, we designed a pair of primers F4 (5′-ATC TCC TAC CTG CAC CCA GGA AAC-3′) and R4 (5′-CTG CAT GGT GTG AGC AAA AGC CT-3′) to amplify part of the *Rbp3* exon 1 sequences (∼1900 bp) (Figure S1B). Details on primers and corresponding bat species are listed in Table S2. For both genes, Polymerase Chain Reactions (PCR) were conducted using Premix Ex Taq^TM^ (TaKaRa) with the following conditions: denaturation at 95°C for 5 min, 32 amplification cycles [95°C for 30 s, annealing temperature (see Table S2) for 30 s, 72°C for 1.5∼2.5 min (depending upon the target length)], and a final extension at 72°C for 10 min. All PCR products were isolated using 1% agarose gels and purified with Gel Extraction Kits (Qiagen), ligated into pGEM-T easy vector (Promega), cloned and sequenced using the Terminator kits (Applied Biosystems) on an ABI 3730 DNA sequencer.

We made great efforts to amplify the *Rbp3* exon 1 sequence for all bat species involved in the *Gja10* analysis with all those above-mentioned *Rbp3* primer pairs. However, we failed to amplify the *Rbp3* sequences from some of these species. One possible reason is that the wing membrane biopsies of many species have been stored in our lab for many years and genomic DNA may undergone a degree of degradation thus increasing the difficulties of amplification of long sequences [28], [29], considering the *Rbp3* exon 1 is longer than 3 kb [20]. Another plausible explanation might be that the *Rbp3* sequences in some (if not all) of these bat species has became less conservative because of the relaxation of evolutionary constraints thus reducing the specificity of our primers.

### Sequence Alignment and Phylogenetic Reconstruction

The open reading frames (ORF) of *Gja10* and *Rbp3* of each bat species were checked separately after reference to the correct ORF with mouse *Gja10* (NM_010289) and *Rbp3* (NM_015745) using MEGA4 [30], respectively. The bat sequences containing insertions, deletions and stop codons, indicative of loss-of-function, were identified as nonfunctional. Then the nonfunctional *Gja10* (15 bat species) and *Rbp3* (nine bat species) nucleotide sequences were aligned separately with mouse *Gja10* and *Rbp3* using ClustalX [31] and checked for accuracy by eye, respectively (Figure S2 and Figure S3). The putatively functional *Gja10* (20 bat species) and *Rbp3* (nine bat species) sequences were aligned with mouse *Gja10* and *Rbp3* using MEGA4 after being translated to deduced amino acids (Figure S4A and Figure S5).

For phylogenetic reconstruction, both the nonfunctional and putatively functional *Gja10* and *Rbp3* nucleotide sequences of bat species were aligned with *Gja10* and *Rbp3* sequences of five mammal outgroups using ClustalX [31] and checked for accuracy by eye. Maximum-likelihood analyses were conducted separately for both genes using RaxML v7.0.4 [32] with the rapid hill-climbing algorithm under the General Time Reversible (GTR) + gamma (Γ) nucleotide substitution model with four discrete rate categories. For both genes, two hundred replicates of RaxML searches were performed with a complete random starting tree and nodal supports were determined by non-parametric bootstrapping with 1,000 RaxML bootstrap replicates.

Besides, Bayesian phylogenetic trees were also reconstructed based on the aligned nucleotide sequences of *Gja10* and *Rbp3* using MrBayes 3.1.2 [33]. The TPM1+Γ and TPM2uf+Γ nucleotide substitution models were selected by jModelTest0.1 [34] for *Gja10* and *Rbp3*, respectively. For each Bayesian analysis, 10,000,000 generations of MCMC were performed with sampling frequency set as every 100th generation. The first 2,000,000 generations were discarded as burn-in, since the standard deviations of split frequencies were stable below 0.01 after 2,000,000 generations of MCMC performances. All other options and priors were the default settings of MrBayes 3.1.2 software.

### Molecular Evolutionary Analyses

For molecular evolutionary analyses, the indels and premature stop codons in nonfunctional *Gja10* and *Rbp3* sequences were removed and sequences were realigned using ClustalX. Besides, as the highly unconservative C-terminal extracellular region (colored red in Figure S4B) would affect the molecular evolutionary analyses (data not shown), the sequences of this region were removed from the dataset of the *Gja10* gene. Phylogenetic topologies of 35 and 18 bat species were used separately for molecular evolutionary analyses based on accepted phylogenetic relationships among the bat species studied [35], [36], [37], [38], [39], [40].

For both genes, we conducted two-ratio models [41], in which the d_N_/d_S_ ratio (termed as omega or ω) was allowed to vary between the background and foreground, to determine the selective pressure changes of *Gja10* and *Rbp3* in bat species with nonfunctional *Gja10* and *Rbp3*, respectively. For *Gja10*, separate models were undertaken with the foreground branch set as branches of species from the family of Hipposideridae and Rhinolophidae [collectively termed as rhinolophids (Superfamily Rhinolophoidea)], vespertilionid bats, *M. fuliginosus* and *D. rotundus* which all contained nonfunctional *Gja10* sequences identified using CODEML in the PAML package [42]. For *Rbp3*, separate models were undertaken with the foreground branch set as branches of rhinolophids, *P. abramus* and *Pteronotus* species which all contain nonfunctional *Rbp3* sequences. For each case, the one-ratio model in which ω was fixed among all branches was performed as the null hypothesis [41]. We also conducted separately modified two-ratio models with the ω value of foreground fixed as 1 (relaxed selection) to the former lineages of bats with nonfunctional *Gja10* and *Rbp3*, respectively, to see if the tested branches have undergone relaxed selection. Notably, for each test, the other nonfunctional *Gja10* and *Rbp3* sequences were removed from the datasets to remove their influence upon the ω value of the background and the new dataset was then realigned and checked each time (e.g., when setting rhinolophids as foreground, the sequences of vespertilionid bats, *M. fuliginosus* and *D. rotundus* were removed from the *Gja10* dataset, and the same methods were applied in other analyses). All the results of alternative and null hypotheses were compared using the likelihood ratio tests (LRTs).

Besides, we also reanalyzed the lineages of bats with nonfunctional *Gja10* and *Rbp3* which were tested by using two-ratio model tests using the “TestBranchDNDS.bf” in the HyPhy package [43] to determine whether bat species with nonfunctional sequences evolved under different selection pressures as compared with species with putatively functional sequences. For both genes, the analysis was performed under the HKY85 nucleotide substitution model selected by the Datamonkey web server (http://www.datamonkey.org/), using the complete site-to-site rate variation model, four rate classes and the default amino acid class model.

## Results

### Genetic Data from Bats

**Figure 1:**
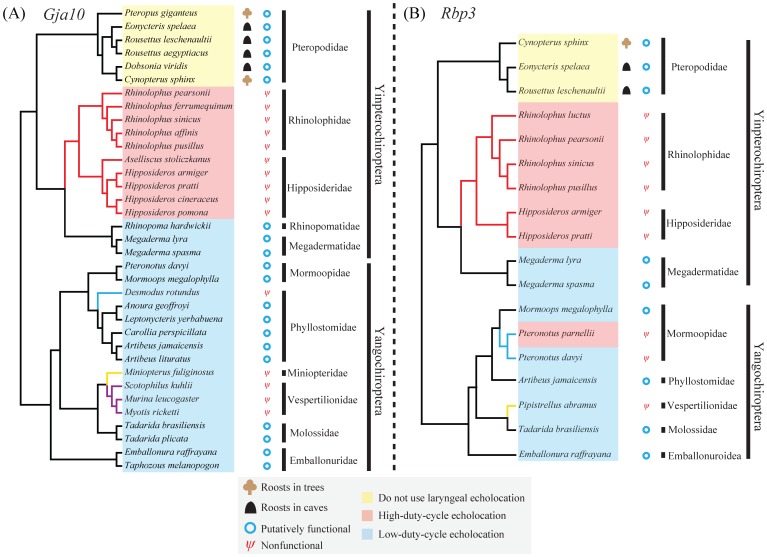
Species topologies of bats showing information on bat phylogeny, gene pseudogenization and echolocation call type. (A) The species topology of 35 bats. Branches leading to rhinolophids, vespertilionid bats, *Miniopterus fuliginosus* and *Desmodus rotundus* which were tested by branch model tests and TestBranchDNDS tests are colored with red, purple, yellow and blue, respectively. (B) The species topology of 18 bats. Branches leading to rhinolophids, *Pteronotus* and *Pipistrellus abramus* which were tested by branch model tests and TestBranchDNDS tests are colored with red, blue and yellow, respectively. Both species topologies are based on accepted bat species relationships (see Materials and Methods for references).

We amplified and sequenced partial coding sequences of two visual perception genes, *Gja10* and *Rbp3*, from a taxonomically wide range of bats. For *Gja10*, we obtained the majority of the exon 2 sequence of the *Gja10* gene from 35 bat species from 11 chiropteran families (Figure 1A and Table S1) [including six species in Pteropodidae without laryngeal echolocation, ten bat species with CF high-duty echolocation calls and 19 species which use low-duty-cycle echolocation typically by emitting frequency-modulated (FM) echolocation calls and separating calls and echoes in the time domain]. The major part of the *Gja10* exon 2 sequences obtained ranged in length from 1168 to 1236 bp (Table S1), accounting for ∼83.5% and ∼81.4% of the exon 2 and the complete coding sequence of the *Gja10* gene, respectively.

For *Rbp3*, we obtained part of the exon 1 sequence of the *Rbp3* gene from 18 bat species from nine chiropteran families (Figure 1B and Table S1) [including three species in Pteropodidae without laryngeal echolocation, seven bat species with CF high-duty echolocation calls and eight species with low-duty-cycle echolocation]. The obtained partial *Rbp3* exon 1 sequences ranged in length from 1844 to 2538 bp (Table S1), accounting for ∼64.3% and ∼59.1% of the exon 1 and the complete coding sequence of *Rbp3* gene, respectively.

### Sequence Alignment and Analyses of *Gja10*

To examine the open reading frame (ORF) of *Gja10* in bats, we aligned the bat sequences with mouse *Gja10*. Alignments showed that the ORF of the sequenced *Gja10* region in six species of Old World fruit bats were all intact (Figure S4A), suggesting that the gene should be functional in these species. However, further sequencing of the complete coding region of the *Gja10* and validation of protein function are still necessary to confirm this.

**Figure 2:**
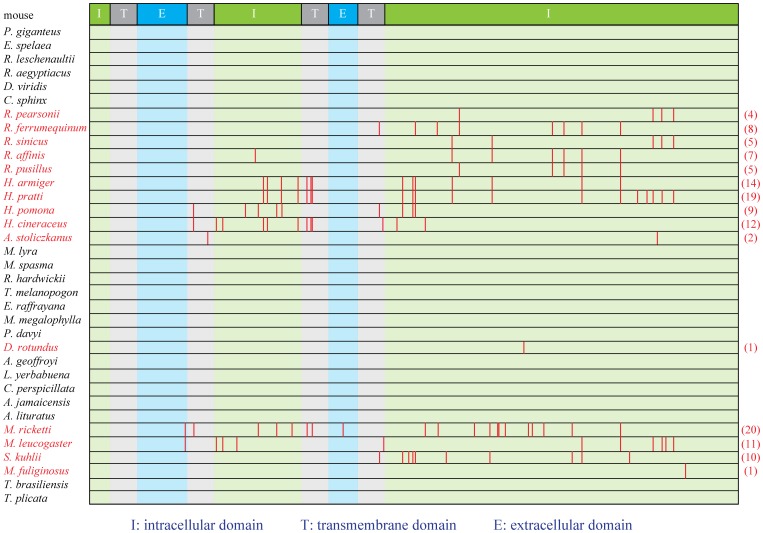
The distribution of premature stop codons along *Gja10* ORF. The protein domains of Gja10 were referred to the prediction of mouse Gja10 from Universal Protein Resource (http://www.uniprot.org/uniprot/Q9WUS4). The 15 bat species with nonfunctional *Gja10* were highlighted in red. Values in parentheses are the total number of premature stop codons. ‘I’, ‘T’ and ‘E’ indicate intracellular domain, transmembrane domain and extracellular domain, respectively. Full species names are presented in Figure 1A.

However in five species of Hipposideridae and five species of Rhinolophidae [collectively termed as rhinolophids (Superfamily Rhinolophoidea)] with CF echolocation calls and high-duty-cycle echolocation, evidence of loss-of-function in *Gja10* was determined, with multiple indels and premature stop codons identified (Figure 2 and Figure S2). Consistent with the results of alignments, our branch model tests showed that the *Gja10* sequences obtained have undergone relaxed selection in rhinolophids (Table 1). Similarly, the results of TestBranchDNDS tests indicated that the d_N_/d_S_ values are significantly different in rhinolophids with nonfunctional *Gja10* as compared with other bat species with putatively functional *Gja10* sequences (*P* < 0.001). Notably, no single indel or premature stop codon is shared by all these bat species (Figure 2 and Figure S2), indicating that the pseudogenization of *Gja10* probably occurred independently in several lineages of rhinolophids.

**Table 1:** Results of branch model tests of selection pressure on the *Gja10* and *Rbp3* genes in bats.

Gene Name	Branches tested and dataset	Model	np^c^	*ℓ*	ω_0_ d	ω_X_ d	Model Compared	*P*
*Gja10*	Rhinolophids	A. One ratio: ω_0_ = ω_OW_	60	−2451.40	0.290	= ω_0_		
	Dataset: 30 sequences	B. Two ratios: ω_0_, ω_OW_	61	−2430.85	0.159	**0.731**	B vs. A	<0.001
	(black and red branches)^a^	C. Two ratios: ω_0,_ ω_OW_ = 1	60	−2432.15	0.160	1	C vs. B	**0.107**
	vespertilionid bats	A. One ratio: ω_0_ = ω_V_	46	−2117.28	0.307	= ω_0_		
	Dataset: 23 sequences	B. Two ratios: ω_0_, ω_V_	47	−2090.36	0.173	**1.446**	B vs. A	<0.001
	(black and purple branches)^a^	C. Two ratios: ω_0,_ ω_V_ = 1	46	−2091.34	0.172	1	C vs. B	**0.163**
	*Desmodus rotundus*	A. One ratio: ω_0_ = ω_D_	42	−1913.35	0.171	= ω_0_		
	Dataset: 21 sequences	B. Two ratios: ω_0_, ω_D_	43	−1913.16	0.168	**0.252**	B vs. A	**0.540**
	(black and blue branches)^a^	C. Two ratios: ω_0,_ ω_D_ = 1	42	−1915.63	0.169	1	C vs. B	0.026
	*Miniopterus fuliginosus*	A. One ratio: ω_0_ = ω_M_	42	−1950.11	0.172	= ω_0_		
	Dataset: 21 sequences	B. Two ratios: ω_0_, ω_M_	43	−1949.88	0.167	**0.235**	B vs. A	**0.502**
	(black and yellow branches)^a^	C. Two ratios: ω_0,_ ω_M_ = 1	42	−1954.66	0.168	1	C vs. B	0.002
*Rbp3*	Rhinolophids	A. One ratio: ω_0_ = ω_OW_	30	−8348.10	0.170	= ω_0_		
	Dataset: 15 sequences	B. Two ratios: ω_0_, ω_OW_	31	−8293.72	0.100	**0.404**	B vs. A	<0.001
	(black and red branches)^b^	C. Two ratios: ω_0,_ ω_OW_ = 1	30	−8328.03	0.098	1	C vs. B	<0.001
								
	*Pipistrellus abramus*	A. One ratio: ω_0_ = ω_Pa_	20	−6686.21	0.141	= ω_0_		
	Dataset: 10 sequences	B. Two ratios: ω_0_, ω_Pa_	21	−6651.60	0.101	**0.438**	B vs. A	<0.001
	(black and yellow branches)^b^	C. Two ratios: ω_0,_ ω_PA_ = 1	20	−6667.57	0.101	1	C vs. B	<0.001
	*Pteronotus*	A. One ratio: ω_0_ = ω_Pt_	22	−7088.59	0.163	= ω_0_		
	Dataset: 11 sequences	B. Two ratios: ω_0_, ω_Pt_	23	−7038.36	0.106	**0.532**	B vs. A	<0.001
	(black and blue branches)^b^	C. Two ratios: ω_0,_ ω_Pt_ = 1	22	−7047.78	0.106	1	C vs. B	<0.001

a See Figure 1A for branch labels.

b See Figure 1B for branch labels.

c np: number of parameters.

d ω_X_ and ω_0_ are the ω values for tested branches (i.e., ω_OW,_ ω_V,_ ω_D_ and ω_M_ for *Gja10*, ω_OW,_ ω_Pa_ and ω_Pt_ for *Rbp3*) and other branches, respectively.

For 19 bat species with low-duty-cycle echolocation, evidence for functional loss of *Gja10* was detected in only five species all from the suborder Yangochiroptera (three species from the Vespertilionidae, *Miniopterus fuliginosus* from the Miniopteridae and *Desmodus rotundus* from the Phyllostomidae) (Figure 1A and Figure S2). Similar to the rhinolophids, no single indel or premature stop codon is found to be shared by all these five bat species, but three deletions were found to be shared by all three species from the Vespertilionidae at positions 201, 275 and 1185, respectively (Figure S2), indicating that the functional loss of *Gja10* may have occurred in the ancestor of the Vespertilionidae. However, further studies are needed to confirm this, because the Vespertilionidae is a large family with more than 400 species [44]. Our branch model tests indicated that the *Gja10* sequences obtained have also undergone relaxed selection in these three species from the Vespertilionidae (Table 1). Corresponding to these results, our TestBranchDNDS tests also revealed that the d_N_/d_S_ values are significantly different in vespertilionid bats as compared with other bat species with putatively functional *Gja10* sequences (*P* < 0.001). For *M. fuliginosus* and *D. rotundus*, only one premature stop codon was found in the *Gja10* sequences, suggesting relatively recent functional losses of *Gja10*. Consistent with this result, branch model tests showed that the d_N_/d_S_ (termed as omega or ω) values for obtained *Gja10* sequences of these two species are both significantly lower than 1 (Table 1). Our TestBranchDNDS tests also confirmed these results, as the d_N_/d_S_ values in *M. fuliginosus* and *D. rotundus* are not significantly different from other bat species with putatively functional *Gja10* sequences (*P* = 0.453 and 0.531 for *M. fuliginosus* and *D. rotundus*, respectively). For the other 14 bat species with low-duty-cycle echolocation, the ORFs of *Gja10* sequences were all intact, indicating that the gene should be functional in these species (see Figure 1A for species and family names). Taken together, our results suggested that the *Gja10* gene was probably functional in the ancestor of bats but has been lost on a number of occasions independently in echolocating bats.

**Figure 3:**
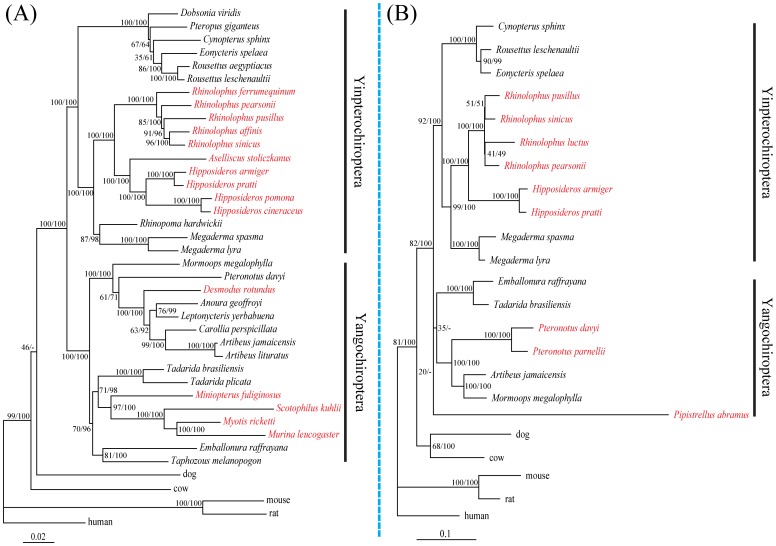
Maximum-likelihood phylogenetic trees based on aligned nucleotide sequences. (A) Maximum-likelihood tree based on the *Gja10* nucleotide sequences under the GTR+Γ nucleotide substitution model. (B) Maximum-likelihood tree based on the *Rbp3* nucleotide sequences under the GTR+Γ nucleotide substitution model. Values on the nodes are maximum-likelihood bootstrap values/Bayesian posterior probabilities. Bat species with nonfunctional *Gja10* and *Rbp3* sequences are highlighted in red, respectively.

Our phylogenetic reconstruction based on the *Gja10* nucleotide sequences revealed a tree in which the major groupings agreed with the accepted species tree (Figure 3A). Both the maximum-likelihood (ML) and the Bayesian analyses highly supported the monophyly of Chiroptera [ML bootstrap 100% and Bayesian posterior probability (BPP) of 100%] (Figure 3A). The species of Pteropodidae grouped with species from the family Rhinolophidae, Hipposideridae, Megadermatidae and Rhinopomatidae to comprise the clade Yinpterochiroptera (100% ML bootstrap and 100% BPP) (Figure 3A). And other bat species from the family Mormoopidae, Phyllostomidae, Vespertilionidae, Miniopteridae, Molossidae and Emballonuridae grouped together and comprised the clade Yangochiroptera (100% ML bootstrap and 100% BPP) (Figure 3A).

### Sequence Alignment and Analyses of *Rbp3*

We also sequenced and studied another visual perception gene *Rbp3* in 18 bat species from nine chiropteran families. After alignment of the bat sequences with mouse *Rbp3*, our results showed that the ORF of the sequenced *Rbp3* region were all intact in three species of Old World fruit bats (Figure S5), indicating that the *Rbp3* gene should also be functional in this lineage of species. Of course, further studies are still necessary to confirm this.

**Figure 4:**
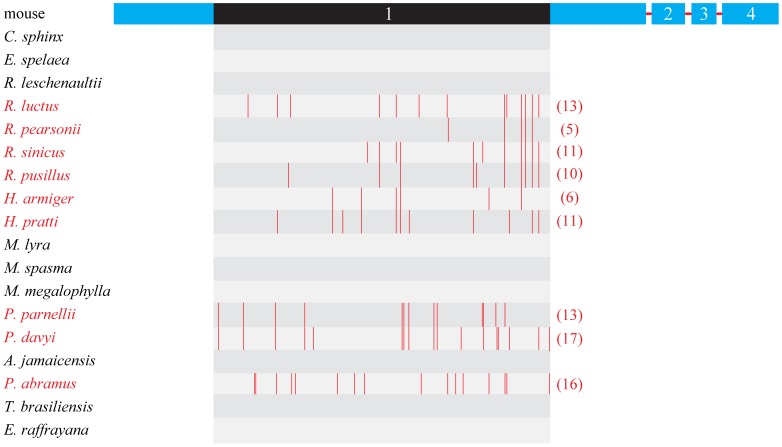
The distribution of premature stop codons along *Rbp3* ORF. Four exons of *Rbp3* are indicated by Arabic numbers. The nine bat species with nonfunctional *Rbp3* were highlighted in red. Values in parentheses are the total number of premature stop codons. The region in exon 1 of *Rbp3* which sequenced and analyzed in the study is highlighted in black. Full species names are presented in Figure 1B.

Similarly, evidence for functional loss of *Rbp3* was also found in rhinolophids, with multiple indels and premature stop codons identified in two species of Hipposideridae and four species of Rhinolophidae (Figure 4 and Figure S3). Indeed, our branch model tests revealed a change in selective pressure on the *Rbp3* gene in this lineage of bats resulting from the relaxation of selective constraints (Table 1). Moreover, the results of TestBranchDNDS tests also indicated that the d_N_/d_S_ values are significantly different in rhinolophids with nonfunctional *Rbp3* as compared with other bat species with putatively functional *Rbp3* sequences (*P* < 0.001). Among the ORF-disrupting mutations, a 17-bp deletion was shared by two species from the family Hipposideridae at position 979 (Figure S3), suggesting the functional loss of *Rbp3* may have occurred in the common ancestor of Hipposideridae. However, similar to that of *Gja10*, no single indel or premature stop codon is shared by all these six bat species (Figure 4 and Figure S3), indicating that the pseudogenization of *Rbp3* probably also occurred independently on several occasions in rhinolophids.

We also obtained part of the exon 1 sequence of the *Rbp3* gene from *P. parnellii* (Mormoopidae), a neotropical bat species which has independently evolved CF echolocation with Doppler shift compensation [45]. Evidence of loss-of-function in *Rbp3* was also detected in this bat species (Figure 4 and Figure S3). However, the loss-of-function of *Rbp3* was also found in *P. davyi*, a close relative of *P. parnellii* with low-duty-cycle echolocation [46]. Besides, five deletions and one insertion were found to be shared by these two species at positions 312, 544, 852, 980, 1905 and 1298, respectively (Figure S3), suggesting that the functional loss of *Rbp3* occurred in the common ancestor of bats in the genus *Pteronotus* before the independent evolution of CF echolocation in *P. parnellii*.

For eight bat species with low-duty-cycle echolocation, evidence for functional loss of *Rbp3* was detected in only two species, including *P. abramus* from the Vespertilionidae and *P. davyi* (mentioned above) from the Mormoopidae (Figure 1B and Figure S3). Consistent with these results, evidence of selective pressure changes of *Rbp3* associated with the relaxation of selective constraints was detected by our branch model tests on branches leading to *P. abramus* and *Pteronotus* (Table 1) and also by the TestBranchDNDS tests (*P* < 0.001 and *P* < 0.001 for *P. abramus* and *Pteronotus*, respectively). As is the case with *Gja10*, no single indel or premature stop codon was shared by these two bat species (Figure S3). The ORFs of *Rbp3* sequences were intact in all the other six bat species with low-duty-cycle echolocation, suggesting that the gene may be functional in these species (Figure S5). As with *Gja10*, our results suggest that the *Rbp3* gene was probably functional in the ancestor of bats but has been lost on a number of occasions independently in echolocating bat lineages.

Similarly, our maximum-likelihood and Bayesian phylogenetic reconstruction analyses based on the *Rbp3* nucleotide sequences also revealed a tree in which the major groupings agreed with the accepted species relationships (Figure 3B). The monophyly of Chiroptera was also highly supported by both methods (82% ML bootstrap and 100% BPP) (Figure 3B). The species of Pteropodidae grouped with species from the family Rhinolophidae, Hipposideridae and Megadermatidae to comprise the clade Yinpterochiroptera (92% ML bootstrap and 100% BPP) (Figure 3B). And other bat species from the family Mormoopidae, Phyllostomidae, Vespertilionidae, Molossidae and Emballonuridae grouped together and comprised the clade Yangochiroptera, however, with relatively low support (20% ML bootstrap and lack of support from BPP) (Figure 3B). This situation appears to stem from accelerated evolution of *Rbp3* caused by relaxation of evolutionary constraints in *P. abramus*. Besides, we found that the species *M. megalophylla* grouped with the species *A. jamaicensis* from the family Phyllostomidae instead of its close relatives, *P. davyi* and *P. parnellii*. This conflict may also be caused by evolutionary constraints relaxation of *Rbp3* in *P. davyi* and *P. parnellii*.

## Discussion

In this study, we sequenced and compared two visual perception genes, *Gja10* and *Rbp3*, in a wide range of bat species with and without laryngeal echolocation. For both genes, a sharply distinct evolutionary trajectory has been found between bat species with and without laryngeal echolocation. Our results showed that both genes were likely intact and putatively functional in species of Old World fruit bats, but have became pseudogenes in the lineage of rhinolophids that emit constant frequency echolocation calls with Doppler shift compensation at high-duty-cycles, and in some bat species that emit echolocation calls at low-duty-cycles.

Considering the lacking of evidence from mRNA expression and protein function, one may speculate that nonfunctional *Gja10* and *Rbp3* sequences of bat species might be from processed pseudogenes which are not orthologous to the other *Gja10* and *Rbp3* sequences and/or from other gene members of the same gene family with high sequence similarity, especially in the *Gja10* gene which belong to the connexin gene family [47]. However, we argue that such possibility is highly unlikely. To date, members of connexin gene family are all well known to be single-copy genes [48], [49], and clear evidence has shown that the *Gja10* gene is a single-copy gene in the mouse [50]. Moreover, the *Rbp3* gene is also known to be a single-copy nuclear gene [25], [51], [52]. Thus it is highly likely that the *Gja10* and *Rbp3* genes should also be single-copy genes in bat lineages. Furthermore, if nonspecific amplifications of other gene member (s) have occured, the inclusion of paralogous gene (s) in the dataset could easily lead to serious conflicts between gene and species trees [53]. However, our phylogenetic reconstruction analyses based on *Gja10* and *Rbp3* nucleotide sequences respectively revealed gene topologies in which the major groupings agreed with the accepted species relationships with high levels of support (Figure 3). Thus, the possibility that the nonfunctional *Gja10* and *Rbp3* sequences of bat species are from other gene members of the same gene family could be ruled out. Taken together, evidence strongly suggested that our amplified nonfunctional *Gja10* and *Rbp3* sequences in some laryngeal echolocating bats are from the same genes that are functional in other bats and mammals but have became pseudogenes because of accumulations of indels caused by relaxation of evolutionary constraints.

Our results showed that both genes were likely intact and putatively functional in species of Old World fruit bats (six and three species for *Gja10* and *Rbp3*, respectively), indicating that these two genes were important to species in this lineage. These results thus are congruent with the fact that Old World fruit bats depend largely on vision for orientation and foraging [54], [55], [56]. Without the capability of laryngeal echolocation, species of Old World fruit bats are known to possess a highly developed visual system [57], [58] and a specialized tapetum lucidum [59] to enhance their visual sensitivity in dim-light environments. Besides, in contrast to the patterns observed for the *Sws1* gene [5], our results showed that both *Gja10* and *Rbp3* were putatively functional in tree roosting and cave roosting Old World fruit bats (Figure 1), indicating that roosting ecology has no effect on these two genes in Old World fruit bats.

In contrast to the nonlaryngeal echolocating Old World fruit bats, functional losses of both *Gja10* and *Rbp3* were found in all rhinolophids that use high-duty-cycle echolocation (ten and six species for *Gja10* and *Rbp3*, respectively). These results indicated that the evolution of sophisticated CF echolocation at high-duty-cycles is related to the extensive investment in neural processing of echoes [60] resulting in less reliance on vision for nocturnal life. By using CF echolocation and combined Doppler shift compensation, rhinolophids can not only efficiently detect but also classify their prey [61]. Thus CF echolocation is considered perhaps the most sophisticated form of nocturnal sensory adaptation within mammals [62]. Indeed, a trade-off between the sensory modalities of vision and hearing in bats using high-duty-cycle echolocation is supported by other emerging molecular evidence. A key hearing gene, *Prestin*, which plays a pivotal role in high frequency sensitivity and selection, has undergone strong positive selection in high-duty-cycle echolocators [63], [64], [65]. In contrast, the short-wavelength opsin gene (*Sws1*) has became a pseudogene presumably as a consequence of the trade-off between investment in hearing and vision in rhinolophids [5].

Bats with low-duty-cycle echolocation showed more complicated evolutionary patterns for both *Gja10* and *Rbp3* than documented for rhinolophids. The putative functionality of *Gja10* and *Rbp3* in most low-duty-cycle echolocating bats indicated that these species may rely more on vision [66], [67]. The rod-dominated eyes [68], [69], [70] of echolocating bats work well under a dim-light environment [71], [72]. Electrophysiological studies revealed that the electrical coupling between horizontal cells is indeed maximized under dim ambient conditions [73], indicating an important role of Gja10 in dim light vision. Moreover, the Irbp also plays an important role in the normal visual cycle with the lack of this protein causing significant reductions in electroretinogram responses of both rods and cones in *Rbp3* ^−/−^ mice [18], [19]. Thus, it is easy to understand why both *Gja10* and *Rbp3* genes have been conserved in most low-duty-cycle echolocating bats. Besides, the pseudogenization of these two visual perception genes (especially the *Gja10* gene, Figure 1A) mainly in rhinolophids and in some species of Vespertilionidae indicates similar evolutionary patterns between these two lineages. Indeed, the morphological parameters of the retina and the estimated visual acuity in species of Rhinolophidae and Vespertilionidae are more similar to each other compared with those of other bat species [74]. It is interesting to note that the *Gja10* gene has also became pseudogene in the common vampire bat, *Desmodus rotundus*, which is argued to have a good visual ability [75]. Many behavioral studies have reported that *D. rotundus* tends to emerge only in complete darkness with its peak activity often occurring in the darkest part of the night [76], [77], [78]. Such behaviors may reduce reliance on dim-light vision which should be important in most insectivorous low-duty-cycle echolocating bats that emerge shortly after sunset [78]. Indeed, electrophysiological studies revealed that the retina of *D. rotundus* has a weak light tolerance [71]. Besides, it is well known that other senses including thermal sensation [79] and olfaction [80] are also involved in foraging behavior by *D. rotundus*. Thus, it is possible that the capability of laryngeal echolocation combined with special behaviors and usages of alternative senses like thermal sensation and olfaction have reduced the reliance on dim-light vision and ultimately caused the functional loss of the *Gja10* gene in *D. rotundus*.

Besides, our results suggested that both the *Gja10* gene and the *Rbp3* gene were probably functional in the ancestor of bats but have been lost on a number of occasions independently in echolocating bats. These results could be explained by either of the two scenarios of the evolution of laryngeal echolocation in bats [60], [81]: 1) laryngeal echolocation was gained once in the ancestral bat but subsequently lost in the ancestor of Pteropodidae; 2) laryngeal echolocation was gained independently in at least two lineages of bats. For the second scenario, it is easy to imagine that the independent evolution of laryngeal echolocation in different lineages of echolocating bats has gradually reduced their dependence on vision for nocturnal life eventually leading to the losses of these two visual perception genes in echolocating bats (i.e. species that emit CF echolocation calls with Doppler shift compensation at high-duty-cycles and some bat species that emit echolocation calls at low-duty-cycles). In the first scenario, it may possible that the vision still played an important role for ancestral bats after the evolution of laryngeal echolocation. Then during the latter evolutionary history of Chiroptera, these two visual perception genes independently became pseudogenes in different lineages of echolocating bats in relation to the trade-off between vision and echolocation, because relaxation of vision should occur after the evolution of echolocation. For the Old World fruit bats, it is plausible that these two genes might still have been under evolutionary constraints in the ancestor of Pteropodidae and the subsequent loss of laryngeal echolocation further enforces the critical role of vision for orientation and foraging at night. Thus, further studies focusing on vision genes, hearing genes interpreted in the context of the fossil record are needed to elucidate the evolutionary relationship between echolocation and vision in bats.

In conclusion, our study provides further evidence for the hypothesis that a trade-off exists between the sensory modalities of vision and hearing in echolocating bats at the genetic level. The surprisingly similar evolutionary patterns found in *Gja10* and *Rbp3* in bats lead us to hypothesize that numerous other visual perception genes will have undergone relaxed selection or even functional loss in echolocating bats, especially in species using high-duty-cycle echolocation where neural investment in auditory processing is substantial. Moreover, our results also highlight that visual scientists must be cautious when selecting species for physiological studies of visual function, because both *Gja10* and *Rbp3* have important roles in normal visual function and show varying levels of pseudogenization in echolocating bats.

## Supporting Information

Figure S1 **Schematic to show primer designations for the** ***Gja10*** **and the** ***Rbp3*** **amplification.** (A) Primer pair for the *Gja10* gene amplification. The protein domains of Gja10 were referred to the prediction of mouse Gja10 from Universal Protein Resource (http://www.uniprot.org/uniprot/Q9WUS4). ‘I’, ‘T’ and ‘E’ indicate intracellular domain, transmembrane domain and extracellular domain, respectively. (B) Primer pairs for the *Rbp3* gene amplification. Four exons of *Rbp3* are indicated by Arabic numbers.
(PDF)

Figure S2 **Alignment of the newly obtained nonfunctional** ***Gja10*** **sequences of 15 bats with mouse** ***Gja10*** **sequences.** Codons in correct open reading frame are indicated by shading. Insertions, deletions and premature stop codons are highlighted with yellow, blue and red boxes, respectively. Full species names are presented in Figure 1A.
(PDF)

Figure S3 **Alignment of the newly obtained nonfunctional** ***Rbp3*** **sequences of 9 bats with mouse** ***Rbp3*** **sequences.** Codons in correct open reading frame are indicated by shading. Insertions, deletions and premature stop codons are highlighted with yellow, blue and red boxes, respectively. Full species name are presented in Figure 1B.
(PDF)

Figure S4 **Alignment of the amino acid sequences of functional bat** ***Gja10*** **gene with mouse** ***Gja10*** **.** (A) Alignment of the functional *Gja10* sequences of 20 bats with mouse *Gja10* sequences. (B) Secondary protein structure of Gja10. The protein structure is based on the prediction of mouse Gja10 from Universal Protein Resource (http://www.uniprot.org/uniprot/Q9WUS4). The highly unconservative C-terminal extracellular region which was removed from the dataset for molecular evolutionary analyses (see Materials and Methods) is indicated by a red line and blue box.
(PDF)

Figure S5 **Alignment of the amino acid sequences of functional bat** ***Rbp3*** **gene with mouse** ***Rbp3*** **(only the variable sites are shown).**
(PDF)

Table S1 **Information on bat species examined for** ***Gja10*** **and** ***Rbp3*** **genes in the study.**
(DOC)

Table S2 **Information on primers used for** ***Gja10*** **and** ***Rbp3*** **sequence amplification.**
(DOC)
