# Supplementary figures and images for: Independent Losses of Visual Perception Genes Gja10 and Rbp3 in Echolocating Bats (Order: Chiroptera)
Source: PLoS One. 2013 Jul 18;8(7):e68867. doi: 10.1371/journal.pone.0068867 (PMC3715546; doi:10.1371/journal.pone.0068867)

(A) *Gja10*

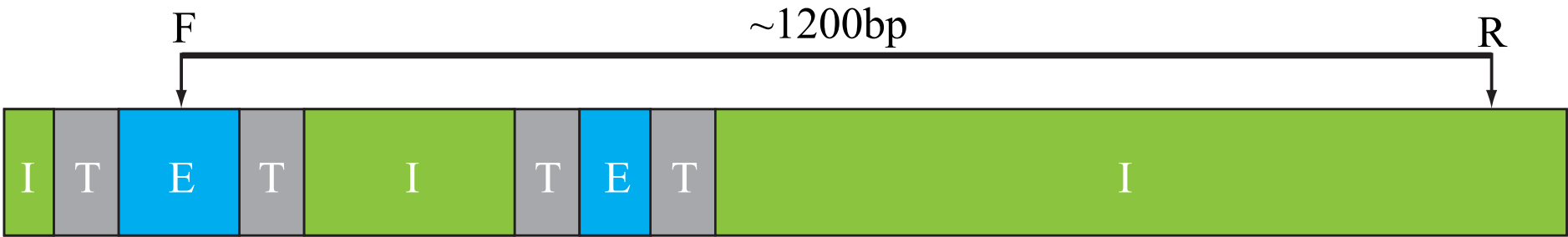

(B) *Rbp3*

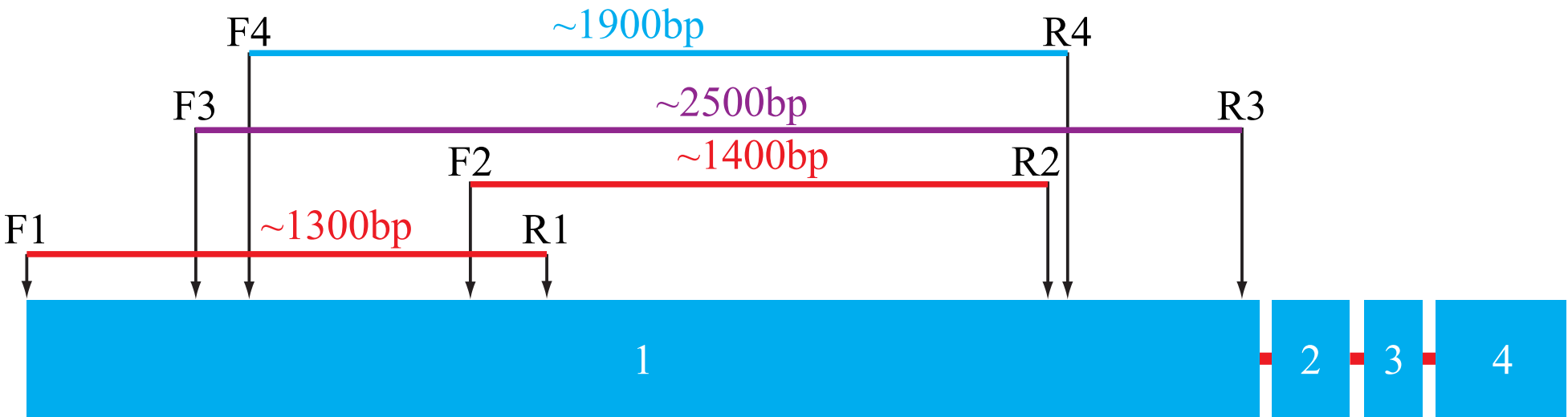

Supplement: Figure S1 — Schematic to show primer designations for the Gja10 and the Rbp3 amplification. (A) Primer pair for the Gja10 gene amplification. The protein domains of Gja10 were referred to the prediction of mouse Gja10 from Universal Protein Resource (http://www.uniprot.org/uniprot/Q9WUS4). ‘I’, ‘T’ and ‘E’ indicate intracellular domain, transmembrane domain and extracellular domain, respectively. (B) Primer pairs for the Rbp3 gene amplification. Four exons of Rbp3 are indicated by Arabic numbers. (PDF) [file pone.0068867.s001.pdf]
