# Supplementary material for: Independent Losses of Visual Perception Genes Gja10 and Rbp3 in Echolocating Bats (Order: Chiroptera)
Source: PLoS One. 2013 Jul 18;8(7):e68867. doi: 10.1371/journal.pone.0068867 (PMC3715546; doi:10.1371/journal.pone.0068867)
Supplement: Figure S2 — Alignment of the newly obtained nonfunctional Gja10 sequences of 15 bats with mouse Gja10 sequences. Codons in correct open reading frame are indicated by shading. Insertions, deletions and premature stop codons are highlighted with yellow, blue and red boxes, respectively. Full species names are presented in Figure 1A. (PDF) [file pone.0068867.s002.pdf]

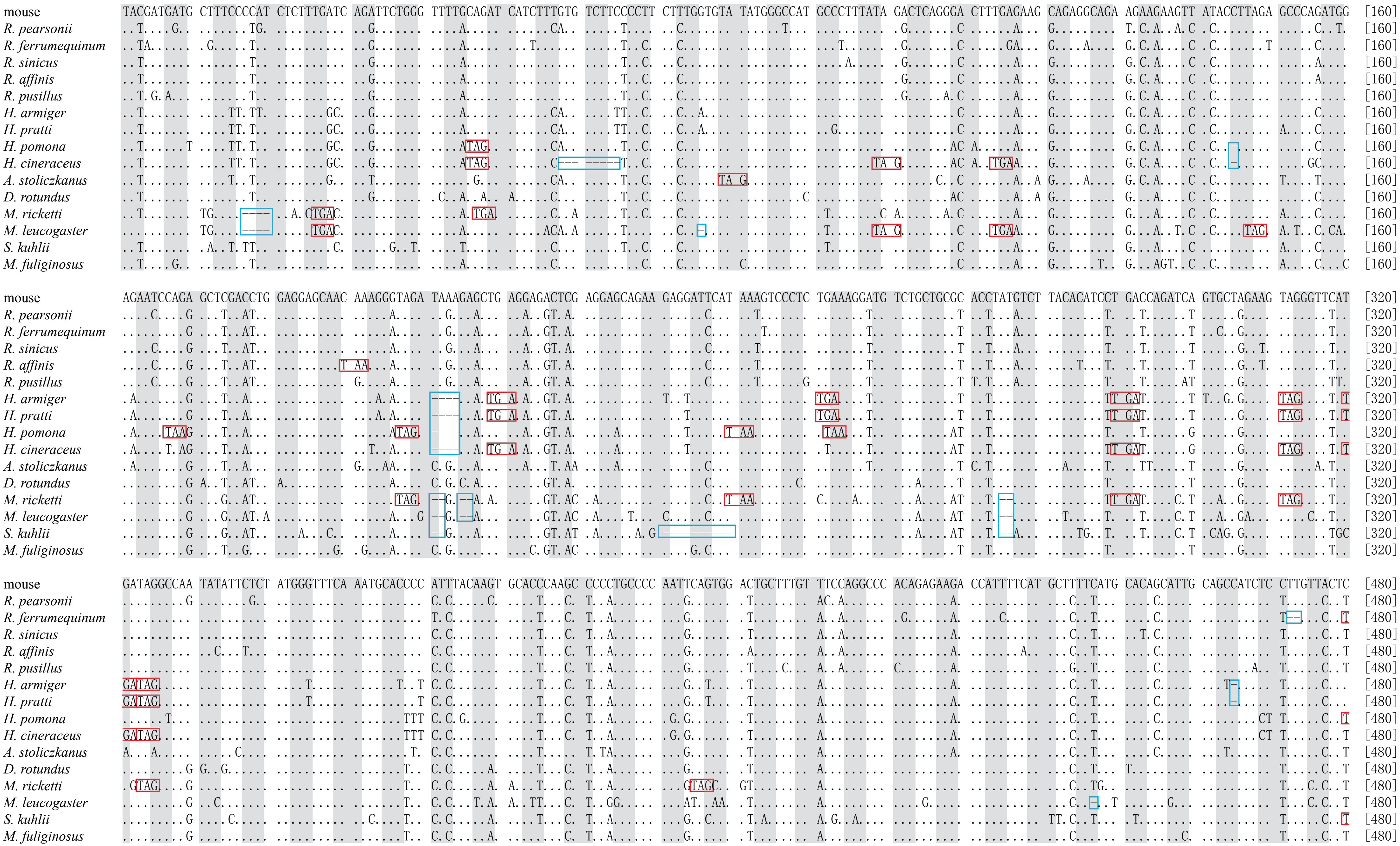

mouse TACGATGATG CTTTCCCCAT CTCTTTGATC AGATTCTGGG TTTTGCAGAT CATCTTTGTG TCTTCCCCTT CTTTGGTGTA TATGGGCCAT GCCCTTTATA GACTCAGGGA CTTTGAGAAG CAGAGGCAGA AGAAGAAGTT ATACCTTAGA GCCCAGATGG [160]
mouse AGAATCCAGA GCTCGACCTG GAGGAGCAAC AAAGGGTAGA TAAAGAGCTG AGGAGACTCG AGGAGCAGAA GAGGATTCAT AAAGTCCCTC TGAAAGGATG TCTGCTGCGC ACCTATGTCT TACACATCCT GACCAGATCA GTGCTAGAAG TAGGGTTCAT [320]
mouse GATAGGCCAA TATATTCTCT ATGGGTTTCA AATGCACCCC ATTTACAAGT GCACCCAAGC CCCCTGCCCC AATTCAGTGG ACTGCTTTGT TTCCAGGCCC ACAGAGAAGA CCATTTTCAT GCTTTTCATG CACAGCATTG CAGCCATCTC CTTGTTACTC [480]
R. pearsonii
R. ferrumequinum
R. sinicus
R. affinis
R. pusillus
H. armiger
H. pratti
H. pomona
H. cineraceus
A. stoliczkanus
D. rotundus
M. ricketti
M. leucogaster
S. kuhlii
M. fuliginosus

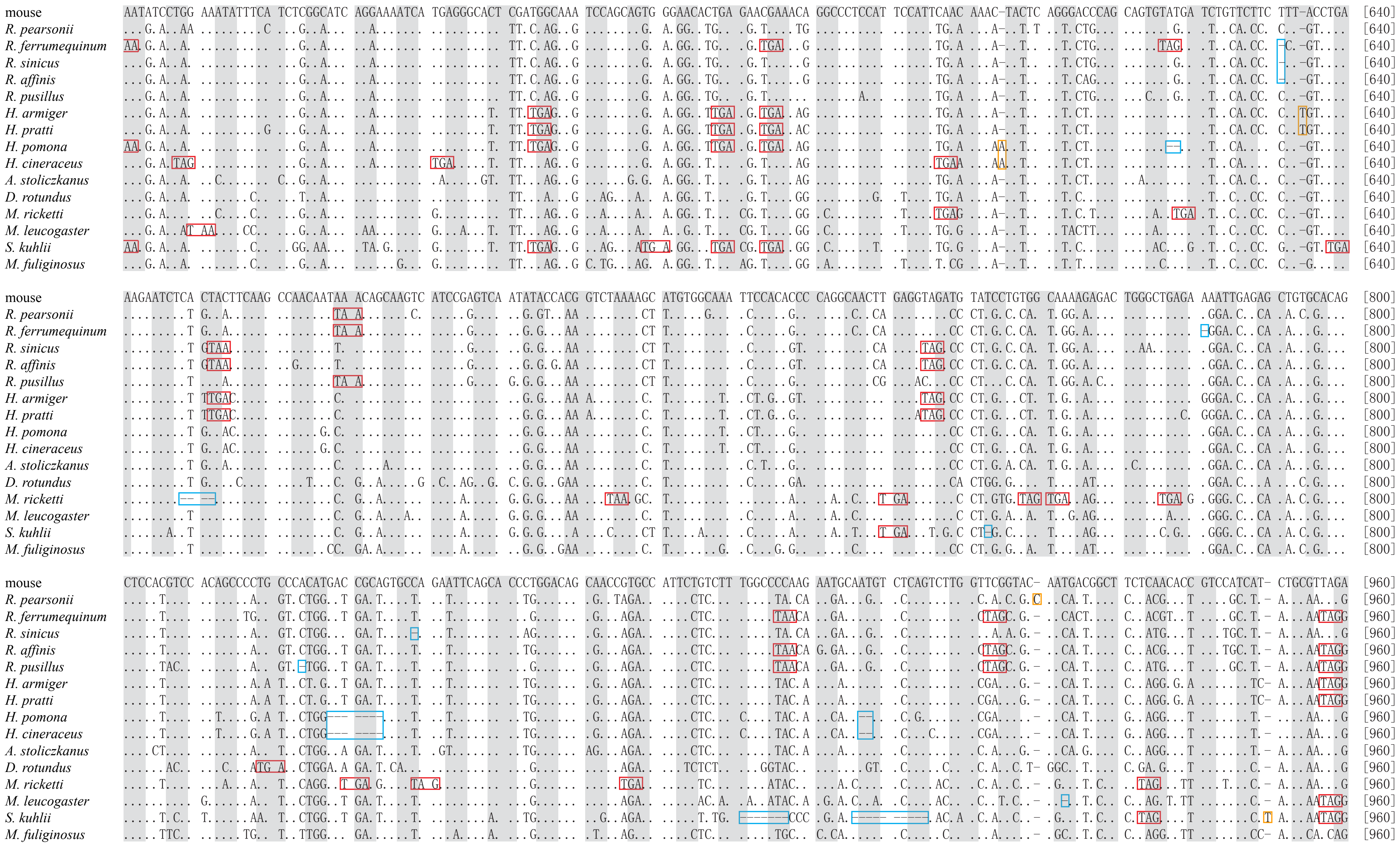

mouse AATATCCTGG AAATATTTCA TCTCGGCATC AGGAAAATCA TGAGGGCACT CGATGGCAAA TCCAGCAGTG GGAACACTGA GAACGAAACA GGCCCTCCAT TCCATTCAAC AAAC-TACTC AGGGACCCAG CAGTGTATGA TCTGTTCTTC TTT-ACCTGA [640]
R. pearsonii
R. ferrumequinum
R. sinicus
R. affinis
R. pusillus
H. armiger
H. pratti
H. pomona
H. cineraceus
A. stoliczkanus
D. rotundus
M. ricketti
M. leucogaster
S. kuhlii
M. fuliginosus
mouse AAGAATCTCA CTACTTCAAG CCAACAATAA ACAGCAAGTC ATCCGAGTCA ATATACCACG GTCTAAAAGC ATGTGGCAAA TTCCACACCC CAGGCAACTT GAGGTAGATG TATCCTGTGG CAAAAGAGAC TGGGCTGAGA AAATTGAGAG CTGTGCACAG [800]
mouse CTCCACGTCC ACAGCCCCTG CCCACATGAC CGCAGTGCCA GAATTCAGCA CCCTGGACAG CAACCGTGCC ATTCTGTCTT TGGCCCCAAG AATGCAATGT CTCAGTCTTG GTTCGGTAC- AATGACGGCT TCTCAACACC GTCCATCAT- CTGCGTTAGA [960]

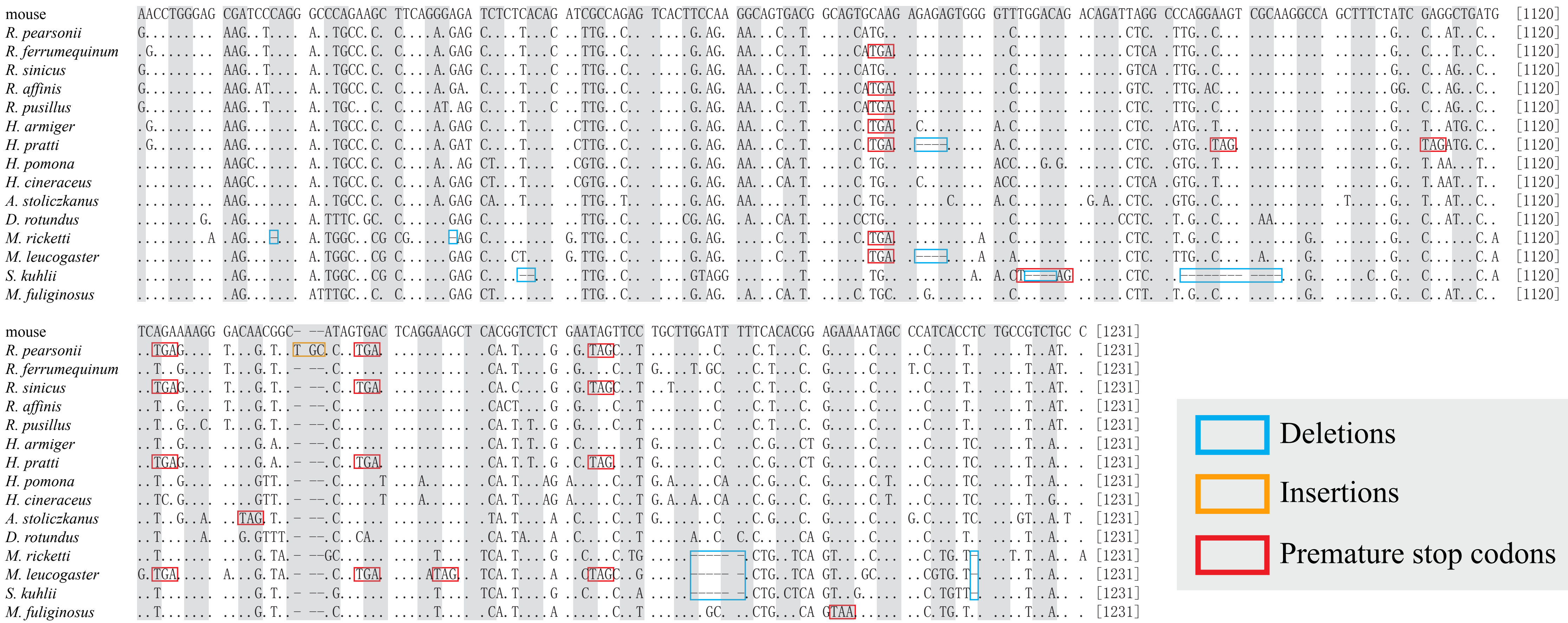
mouse
R. pearsonii
R. ferrumequinum
R. sinicus
R. affinis
R. pusillus
H. armiger
H. pratti
H. pomona
H. cineraceus
A. stoliczkanus
D. rotundus
M. ricketti
M. leucogaster
S. kuhlii
M. fuliginosus
[1120]
[1231]
Deletions
Insertions
Premature stop codons
