# Supplementary material for: Independent Losses of Visual Perception Genes Gja10 and Rbp3 in Echolocating Bats (Order: Chiroptera)
Source: PLoS One. 2013 Jul 18;8(7):e68867. doi: 10.1371/journal.pone.0068867 (PMC3715546; doi:10.1371/journal.pone.0068867)
Supplement: Figure S3 — Alignment of the newly obtained nonfunctional Rbp3 sequences of 9 bats with mouse Rbp3 sequences. Codons in correct open reading frame are indicated by shading. Insertions, deletions and premature stop codons are highlighted with yellow, blue and red boxes, respectively. Full species name are presented in Figure 1B. (PDF) [file pone.0068867.s003.pdf]

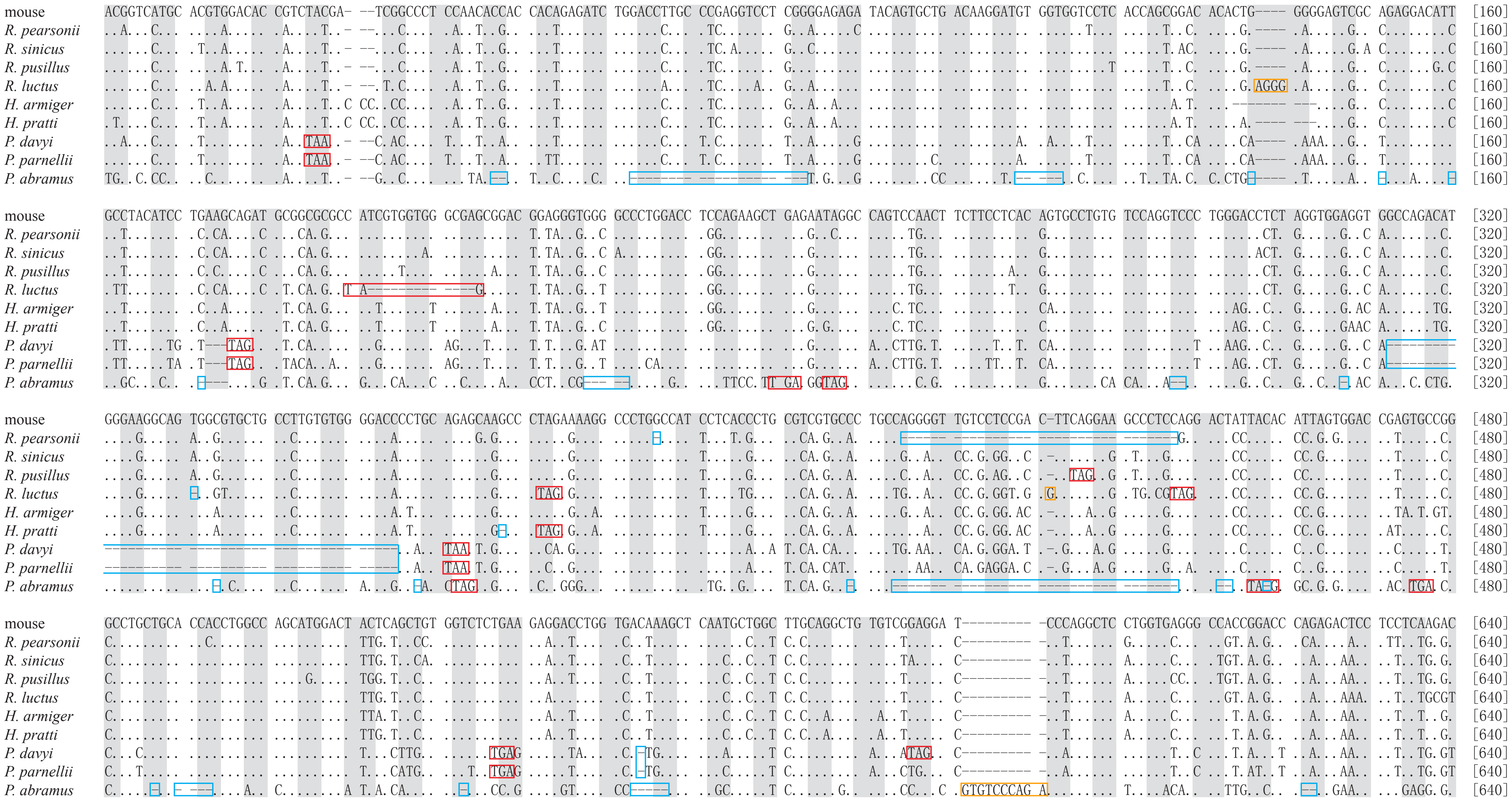
mouse
R. pearsonii
R. sinicus
R. pusillus
R. luctus
H. armiger
H. pratti
P. davyi
P. parnellii
P. abramus
[160]
[320]
[480]
[640]

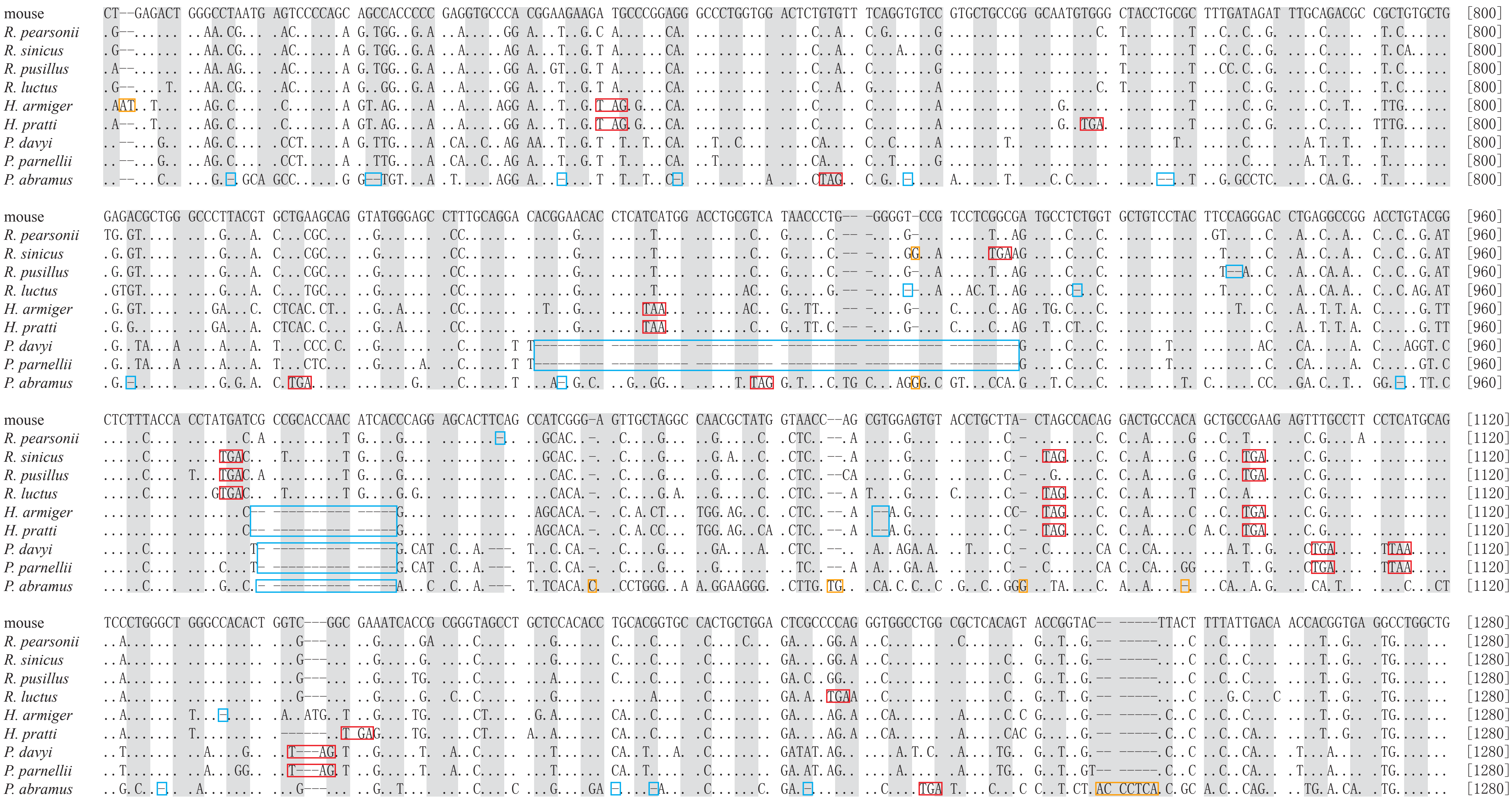

mouse CT--GAGACT GGGCCTAATG AGTCCCCAGC AGCCACCCCC GAGGTGCCCA CGGAAGAAGA TGCCCGGAGG GCCCTGGTGG ACTCTGTGTT TCAGGTGTCC GTGCTGCCGG GCAATGTGGG CTACCTGCGC TTTGATAGAT TTGCAGACGC CGCTGTGCTG [800]
R. pearsonii
R. sinicus
R. pusillus
R. luctus
H. armiger
H. pratti
P. davyi
P. parnellii
P. abramus
mouse GAGACGCTGG GCCCTTACGT GCTGAAGCAG GTATGGGAGC CTTTGCAGGA CACGGAACAC CTCATCATGG ACCTGCGTCA TAACCCTG-- -GGGGT-CCG TCCTCGGCGA TGCCTCTGGT GCTGTCCTAC TTCCAGGGAC CTGAGGCCGG ACCTGTACGG [960]
mouse CTCTTTACCA CCTATGATCG CCGCACCAAC ATCACCCAGG AGCACTTCAG CCATCGGG-A GTTGCTAGGC CAACGCTATG GTAACC--AG CGTGGAGTGT ACCTGCTTA- CTAGCCACAG GACTGCCACA GCTGCCGAAG AGTTTGCCTT CCTCATGCAG [1120]
mouse TCCCTGGGCT GGGCCACACT GGTC---GGC GAAATCACCG CGGGTAGCCT GCTCCACACC TGCACGGTGC CACTGCTGGA CTCGCCCCAG GGTGGCCTGG CGCTCACAGT ACCGGTAC-- -----TTACT TTTATTGACA ACCACGGTGA GGCCTGGCTG [1280]

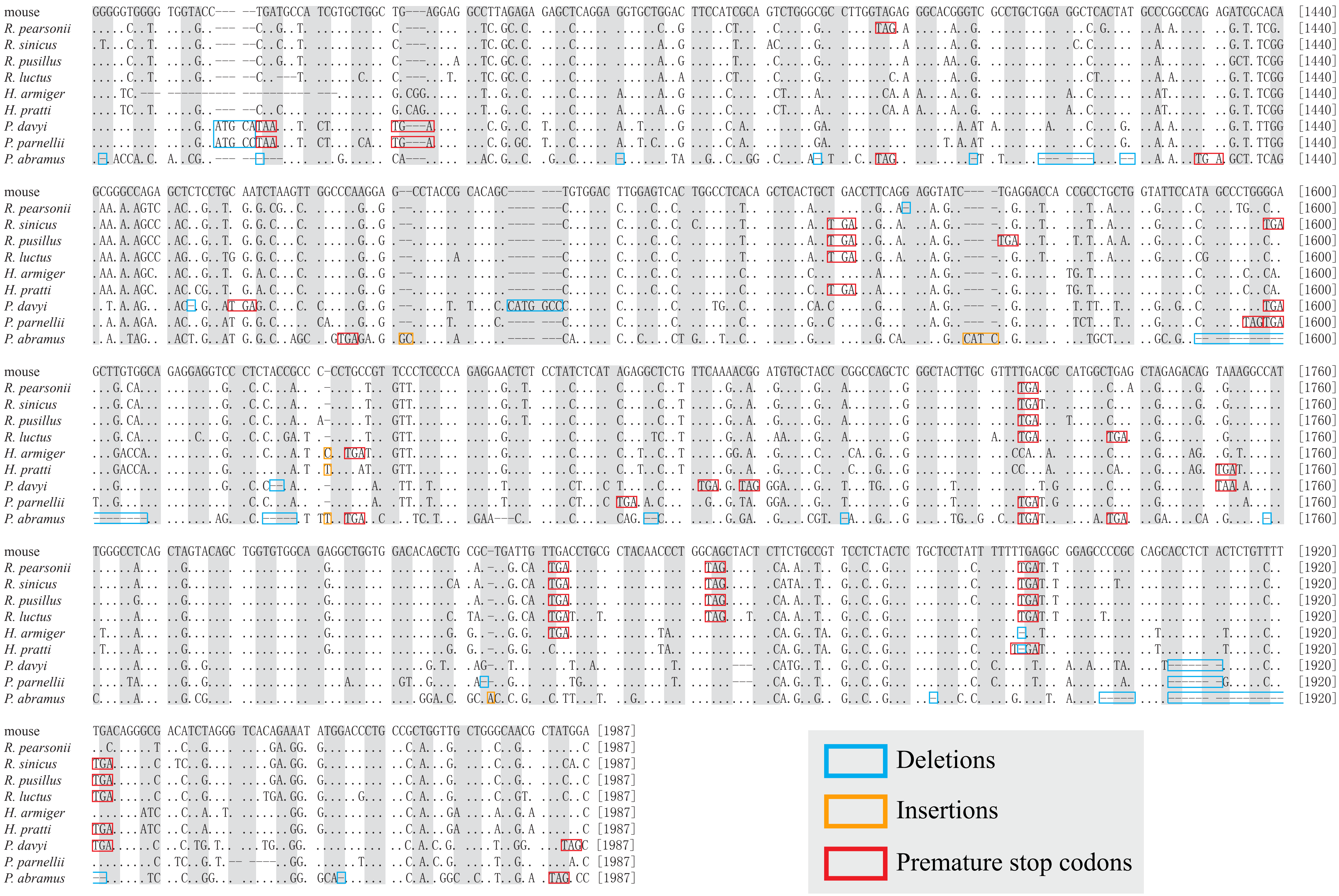
mouse
R. pearsonii
R. sinicus
R. pusillus
R. luctus
H. armiger
H. pratti
P. davyi
P. parnellii
P. abramus
Deletions
Insertions
Premature stop codons
