# Supplementary material for: Independent Losses of Visual Perception Genes Gja10 and Rbp3 in Echolocating Bats (Order: Chiroptera)
Source: PLoS One. 2013 Jul 18;8(7):e68867. doi: 10.1371/journal.pone.0068867 (PMC3715546; doi:10.1371/journal.pone.0068867)
Supplement: Figure S4 — Alignment of the amino acid sequences of functional bat Gja10 gene with mouse Gja10 . (A) Alignment of the functional Gja10 sequences of 20 bats with mouse Gja10 sequences. (B) Secondary protein structure of Gja10. The protein structure is based on the prediction of mouse Gja10 from Universal Protein Resource (http://www.uniprot.org/uniprot/Q9WUS4). The highly unconservative C-terminal extracellular region which was removed from the dataset for molecular evolutionary analyses (see Materials and Methods) is indicated by a red line and blue box. (PDF) [file pone.0068867.s004.pdf]

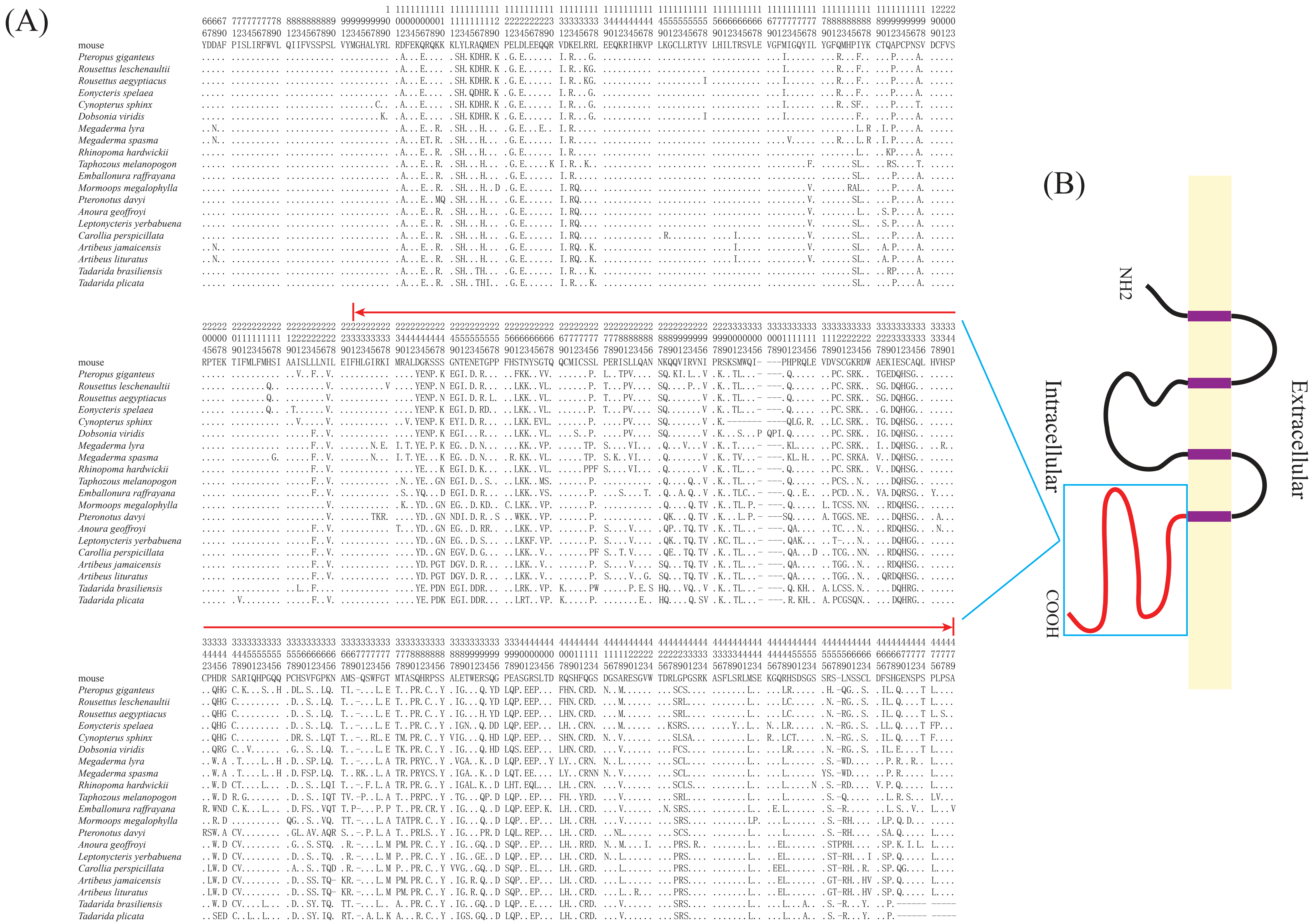
(A)
mouse
Pteropus giganteus
Rousettus leschenaultii
Rousettus aegyptiacus
Eonycteris spelaea
Cynopterus sphinx
Dobsonia viridis
Megaderma lyra
Megaderma spasma
Rhinopoma hardwickii
Taphozous melanopogon
Emballonura raffrayana
Mormoops megalophylla
Pteronotus davyi
Anoura geoffroyi
Leptonycteris yerbabuena
Carollia perspicillata
Artibeus jamaicensis
Artibeus lituratus
Tadarida brasiliensis
Tadarida plicata
(B)
NH2
Intracellular
Extracellular
COOH
