# Supplementary material for: Independent Losses of Visual Perception Genes Gja10 and Rbp3 in Echolocating Bats (Order: Chiroptera)
Source: PLoS One. 2013 Jul 18;8(7):e68867. doi: 10.1371/journal.pone.0068867 (PMC3715546; doi:10.1371/journal.pone.0068867)
Supplement: Figure S5 — Alignment of the amino acid sequences of functional bat Rbp3 gene with mouse Rbp3 (only the variable sites are shown). (PDF) [file pone.0068867.s005.pdf]

	1112222222	2222222222	2222233333	3333333333	3333333333	3333333333	4444444444	4444444444	4444444444
	9990111234	4445555667	7899900222	3333344455	5556667778	8999999999	0000000011	1111224445	5555667778
	3496567332	3490348053	5813527458	0125823923	5790191486	9013456789	1345678901	2568124892	3578080480
mouse	VMVTEVLAGI	AYMRIVREDQ	NPGGTLTRAG	VLREQTLGHH	AMYSAVKAAL	ATGRDSSSRP	TPNESPAATP	ETEDRARAAE	TGYVKDEMHP
Rousettus leschenaultii	ILITQVLAGI	AYMRIVRVDQ	DPGSTLTRAG	MRREQTLAHH	AMFAAVKAAL	AIRRETTSGP	AADYPPEVVP	AEDVQARASG	AGYIHDEMQP
Eonycteris spelaea	ILITQVLAGI	AYMRIVRVDQ	DPGSTLTRAG	MRREQTLAHH	AMFAAVKAAL	AIRRETSSGP	AADYLPEVVP	AEDVQARASG	AGYIHGEMQP
Cynopterus sphinx	ILITQVLAGI	AYMRIVRIDQ	DPGSTLTRAG	MRHEQTLAHH	AMFAAVKAAL	AIRRETSSGP	ATDYLPEVVP	AEDVQARASG	EGYIHDEMQP
Megaderma lyra	ILIMQILAGI	TYMRIVRVDQ	DTGSTLTKAG	LQREQTLAQH	AMFTAVKTAM	AVRKETSSGR	ATNDPPEVAP	AEDVQTRASE	VGHIRDDMQP
Megaderma spasma	ILIVQILAGI	TYMRIVRVDQ	DPGSTLTKAG	LQREQTLAQH	AMFTAVKTDM	AIGKETSSGR	ATNDPPEVAP	AEDVQTRASE	AGHIRDDMQP
Emballonura raffrayana	VLITQVPVAM	AHMRIVQVEQ	DPRSVPTRSG	VRRGRTRAHR	ATFSTVQAAL	VIRKEASPGP	AADDLPEATR	AEDAQGSESG	AGYIRDEMQP
Mormoops megalophylla	VLITQVLAAI	AYMHTVWVDQ	DPGSMLTRAE	MWHEQTLAHH	IMFSMIKAAL	PIRKETSSRP	AADDLREVGP	AEDVQARASG	TSYIRDELQT
Artibeus jamaicensis	VLITQVLAGI	AYKRTIRMDH	DPGSTLIRAE	MRHEQTLAHH	AMFTMIKAAL	PIRKETSSGP	AADDLPEVAP	TEDVQARAPG	ASYIRDELQT
Tadarida brasiliensis	VLVTQVPAAI	AHMRIVQVEQ	DPRSVPTRAG	VRREQARAHH	ATFSTVKTAL	VLRKETSPGP	AADDPPEATP	AEDAQASVSG	AGCIRDEMQP

	4444444555	5555555555	5555555555	5566666666	6666666666	6667777777	7777777778	8888888888	8
	8889999011	2223355577	7778888888	8902223344	4555555666	7990111123	4466677890	0011122233	3
	5790689046	1271208904	5681234568	9932392302	3456789034	8137138915	0115927960	4507916701	3
mouse	SMLVGEAGIQ	HRQNQFGWST	CTPDSPQGGA	LNAERDRSGG	RIAQRARAQS	EVEVVLVPPD	QLQLRDISSE	RQVRTILVQR	G
Rousettus leschenaultii	SVLLGDSGVQ	RTQSQLGWST	HTSETPESGV	LNCDKERSGG	RVVGQTGDRA	DMGAVPIFPE	QLQLKDVTSE	RQVRTVQVQR	G
Eonycteris spelaea	SVLLGDSGVQ	RTQSQLGWST	HTPETPESGV	LNCDKERSGG	HVVEQTGDRA	DMGAVPIFPE	QLQLKDVTSE	RQVRTVQVQR	G
Cynopterus sphinx	SVLLGDSSVQ	RTQSQLGWST	HTPETPESGV	LNCDKERSGG	RVVGQIGDRA	DMGVVPIFPE	QLQLKDVSSE	RQVRTVQVQR	G
Megaderma lyra	SVLLGDTGVQ	RTQSQLSWST	CTPETPEGSV	LNCDRECNGG	RVVGQTGDRA	DMGAVPIFPE	QLQLGDVTSD	RQVRTVQVQS	G
Megaderma spasma	PVILGDTGVQ	RTQSQLSWST	CMPDTLEGSV	LNCDRECNGG	RVVGQTGDRA	DMGAVLIFHE	QLQLGVVTTD	RQIRTVQVQS	G
Emballonura raffrayana	SVLLSGASVQ	RTPAQLGWSS	HAPETPDGGE	LNCDRERGGS	RVVMQTGDQA	EMGTVPVSPE	RVQRRDVTSE	RQVNTVQVQR	G
Mormoops megalophylla	SVLLGEASVR	CMQSQLGWNT	HTPVTAEGGA	FNCEKERRDG	HVVGRTGDRA	EMGEMPVFPE	QLQLRDVTSE	LQVTMVQLQR	S
Artibeus jamaicensis	SVLLGEASVQ	CTQSHLGWST	HTPDTPEGGA	LDCDKERSGG	RVVGRTGDRA	EMGALPIFPE	PLQMRDVTSE	LQVTTVQLQR	S
Tadarida brasiliensis	SVLLSGASVQ	RTPSQLGRST	HTPKTPEGGA	LNCDRERSGG	RVVGQIGDQA	EMGAVPVFPE	RLKLRDVESE	RRVRTVQVRR	G
