# Supplementary material for: Independent Losses of Visual Perception Genes Gja10 and Rbp3 in Echolocating Bats (Order: Chiroptera)
Source: PLoS One. 2013 Jul 18;8(7):e68867. doi: 10.1371/journal.pone.0068867 (PMC3715546; doi:10.1371/journal.pone.0068867)
Supplement: Table S1 — Information on bat species examined for Gja10 and Rbp3 genes in the study. (DOC) [file pone.0068867.s006.doc]

**Table S1. Information on bat species examined for *Gja10* and *Rbp3* genes in the study**

| **Species name** | **Family** | ***Gja10*** | **Sequence length (bp)** | ***Rbp3*** | **Sequence length (bp)** |
| --- | --- | --- | --- | --- | --- |
| *Cynopterus sphinx* | Pteropodidae | KC211191 | 1203 | KC211223 | 2475 |
| *Rousettus leschenaultii* | Pteropodidae | KC211203 | 1224 | KC211229 | 2475 |
| *Eonycteris spelaea* | Pteropodidae | KC211194 | 1224 | KC211225 | 2475 |
| *Pteropus giganteus* | Pteropodidae | KC211200 | 1224 |  |  |
| *Rousettus aegyptiacus* | Pteropodidae | KC211202 | 1224 |  |  |
| *Dobsonia viridis* | Pteropodidae | KC211192 | 1236 |  |  |
| *Rhinolophus ferrumequinum* | Rhinolophidae | KC211217 | 1220 |  |  |
| *Rhinolophus pusillus* | Rhinolophidae | KC211219 | 1223 | KC211238 | 2471 |
| *Rhinolophus sinicus* | Rhinolophidae | KC211220 | 1222 | KC211239 | 2476 |
| *Rhinolophus affinis* | Rhinolophidae | KC211216 | 1223 |  |  |
| *Rhinolophus pearsonii* | Rhinolophidae | KC211218 | 1228 | KC211237 | 2440 |
| *Rhinolophus luctus* | Rhinolophidae |  |  | KC211236 | 2459 |
| *Hipposideros cineraceus* | Hipposideridae | KC211210 | 1203 |  |  |
| *Hipposideros armiger* | Hipposideridae | KC211209 | 1220 | KC211231 | 2438 |
| *Hipposideros pratti* | Hipposideridae | KC211212 | 1216 | KC211232 | 2454 |
| *Hipposideros pomona* | Hipposideridae | KC211211 | 1209 |  |  |
| *Aselliscus stoliczkanus* | Hipposideridae | KC211207 | 1224 |  |  |
| *Megaderma lyra* | Megadermatidae | KC211196 | 1224 | KC211226 | 2538 |
| *Megaderma spasma* | Megadermatidae | KC211197 | 1227 | KC211227 | 2538 |
| *Rhinopoma hardwickii* | Rhinopomatidae | KC211201 | 1224 |  |  |
| *Mormoops megalophylla* | Mormoopidae | KC211198 | 1224 | KC211228 | 1977 |
| *Pteronotus davyi* | Mormoopidae | KC211199 | 1224 | KC211234 | 1855 |
| *Pteronotus parnellii* | Mormoopidae |  |  | KC211235 | 1844 |
| *Desmodus rotundus* | Phyllostomidae | KC211208 | 1224 |  |  |
| *Artibeus jamaicensis* | Phyllostomidae | KC211188 | 1221 | KC211222 | 1977 |
| *Artibeus lituratus* | Phyllostomidae | KC211189 | 1221 |  |  |
| *Leptonycteris yerbabuena* | Phyllostomidae | KC211195 | 1221 |  |  |
| *Anoura geoffroyi* | Phyllostomidae | KC211187 | 1227 |  |  |
| *Carollia perspicillata* | Phyllostomidae | KC211190 | 1224 |  |  |
| *Scotophilus kuhlii* | Vespertilionidae | KC211221 | 1168 |  |  |
| *Myotis ricketti* | Vespertilionidae | KC211215 | 1201 |  |  |
| *Murina leucogaster* | Vespertilionidae | KC211214 | 1200 |  |  |
| *Pipistrellus abramus* | Vespertilionidae |  |  | KC211233 | 2304 |
| *Miniopterus fuliginosus* | Miniopteridae | KC211213 | 1224 |  |  |
| *Tadarida brasiliensis* | Molossidae | KC211204 | 1191 | KC211230 | 1977 |
| *Tadarida plicata* | Molossidae | KC211205 | 1191 |  |  |
| *Taphozous melanopogon* | Emballonuridae | KC211206 | 1224 |  |  |
| *Emballonura raffrayana* | Emballonuridae | KC211193 | 1224 | KC211224 | 1977 |
| human | Hominidae | NM_032602 |  | NM_002900 |  |
| mouse | Muridae | NM_010289 |  | NM_015745 |  |
| rat | Muridae | NM_001173508 |  | NM_001191832 |  |
| cow | Bovidae | XM_001787431 |  | NM_174164 |  |
| dog | Canidae | XM_003639398 |  | XM_546201 |  |
