# Supplementary material for: Independent Losses of Visual Perception Genes Gja10 and Rbp3 in Echolocating Bats (Order: Chiroptera)
Source: PLoS One. 2013 Jul 18;8(7):e68867. doi: 10.1371/journal.pone.0068867 (PMC3715546; doi:10.1371/journal.pone.0068867)
Supplement: Table S2 — Information on primers used for Gja10 and Rbp3 sequence amplification. (DOC) [file pone.0068867.s007.doc]

**Table S2. Information on primers used for *Gja10* and *Rbp3* sequence amplification**

| **Gene Name** | **Species name** | **Primers pairs for PCR** | | **Tm(**°C**)**^b^ |
| --- | --- | --- | --- | --- |
| *Gja10* | all bat species^a^ | Forward primer: F: 5’-CAGCCAGGTTGCAACAATATCTG-3’  Reverse primer: R: 5’-CTTACCATTGATGTTCTGTGCCCA-3’ | | 60 |
| *Rbp3* | *Cynopterus sphinx*  *Rousettus leschenaultii*  *Eonycteris spelaea*  *Rhinolophus luctus*  *Rhinolophus pearsonii*  *Rhinolophus pusillus*  *Rhinolophus sinicus*  *Hipposideros armiger*  *Hipposideros pratti* | Section 1 | Forward primer: F1: 5’-ATGACAAGAGAATGGGCCCTGCTC-3’  Reverse primer: R1: 5’-TGGAAAACGGAGTCCACTAGGGC-3’ | 64 |
|  |  | Section 2 | Forward primer: F2: 5’-ACGATCTGGTCACTAAGCTCAACG-3’  Reverse primer: R2: 5’-ATCAGGATGTAGAGGTCCTTGTGG-3’ | 60 |
|  | *Megaderma lyra*  *Megaderma spasma*  *Pipistrellus abramus* | Forward primer: F3: 5’-AGCCAGGAGGTGGTGAGCAAGTT-3’  Reverse primer: R3: 5’-GGAATCTGGGCTGTCTTCAGGTGT-3’ | | 62 |
|  | *Mormoops megalophylla*  *Pteronotus davyi*  *Pteronotus parnellii*  *Artibeus jamaicensis*  *Tadarida brasiliensis*  *Emballonura raffrayana* | Forward primer: F4: 5’-ATCTCCTACCTGCACCCAGGAAAC-3’  Reverse primer: R4: 5’-CTGCATGGTGTGAGCAAAAGCCT-3’ | | 62 |

^a^see Table S1 for species names.

^b^Tm, annealing temperature.
